# Understanding the Pathogenesis of Kawasaki Disease by Network and Pathway Analysis

**DOI:** 10.1155/2013/989307

**Published:** 2013-03-06

**Authors:** Yu-wen Lv, Jing Wang, Ling Sun, Jian-min Zhang, Lei Cao, Yue-yue Ding, Ye Chen, Ji-juan Dou, Jie Huang, Yi-fei Tang, Wen-tao Wu, Wei-rong Cui, Hai-tao Lv

**Affiliations:** ^1^Department of Pediatric Cardiology, Children's Hospital of Soochow University, Suzhou 215003, China; ^2^Center for Systems Biology, Soochow University, Suzhou 215006, China

## Abstract

Kawasaki disease (KD) is a complex disease, leading to the damage of multisystems. The pathogen that triggers this sophisticated disease is still unknown since it was first reported in 1967. To increase our knowledge on the effects of genes in KD, we extracted statistically significant genes so far associated with this mysterious illness from candidate gene studies and genome-wide association studies. These genes contributed to susceptibility to KD, coronary artery lesions, resistance to initial IVIG treatment, incomplete KD, and so on. Gene ontology category and pathways were analyzed for relationships among these statistically significant genes. These genes were represented in a variety of functional categories, including immune response, inflammatory response, and cellular calcium ion homeostasis. They were mainly enriched in the pathway of immune response. We further highlighted the compelling immune pathway of NF-AT signal and leukocyte interactions combined with another transcription factor NF-**κ**B in the pathogenesis of KD. STRING analysis, a network analysis focusing on protein interactions, validated close contact between these genes and implied the importance of this pathway. This data will contribute to understanding pathogenesis of KD.

## 1. Introduction

KD is a systemic vascular disease preferentially occurring in infants and children [1, 2]. It is characterized by the development of coronary artery aneurysms (CAA) which may result in fatal thrombosis and sudden cardiac failure. Clinical manifestations of KD include prolonged fever (1-2 weeks, mean 10-11 days), conjunctival infection, oral lesions, polymorphous skin rashes, extremity changes, and cervical lymphadenopathy, all of which comprise diagnostic criteria [3]. However, great majority of children failed to manifest typical characteristics. In addition to the diagnostic criteria, there are a broad range of nonspecific clinical features, including irritability, uveitis, aseptic meningitis, cough, vomiting, diarrhea, abdominal pain, gallbladder hydrops, urethritis, arthralgia, arthritis, hypoalbuminemia [4], liver function impairment, and heart failure [5, 6]. The peaked incidence at 9 to 11 months of age coincides with fading of maternal immunity, and symptoms partly similar to other infectious disorders suggest that some microorganisms may trigger this disease. Despite great efforts to identify the cause for nearly a half a century, the etiology of KD still remains unknown [7]. However, the role of genetic susceptibility to KD has long been evident through its striking predilection for children of Japanese ethnicity regardless of their country of residence; compared with Caucasian children, Japanese children have a relative risk of KD that is 10 to 15 times higher [8–10]. Siblings of KD children have a relative risk that is 6 to 10 times greater than that of children without a family history, and the parents of Japanese children with KD are twice as likely to have had KD themselves as children than other adults in the general Japanese population [11–14]. 

Candidate gene studies and genome-wide studies have been successively applied to explore the association between genetic effect and this mysterious disease [15, 16]. Many suspicious genes related to innate and acquired immune functions or to vascular remodeling have been studied [15, 17–19]. Genetic studies of KD were conducted not only to clarify the genetic background but also in the hope of providing clues about its etiology and pathogenesis. However, none of these studies have analyzed the internal association between these significant association genes and explored the possible pathogenic process in KD from overall level.

In this paper, we aim to extract statistically significant genes associated with KD (Up to September 2012, from all English databases) to explore their association and analyze their function in the pathogenesis of KD. This study is a systematic summary of previous research. Further studies on clinical validation will be summarized in our next study.

## 2. Methods and Materials

### 2.1. Extracting Genes with Statistical Significance

We performed a computerized search of Ovid, Google Scholar, and PubMed databases up to September 2012 and reviewed cited references to identify the relevant studies. Citations were screened at the title/abstract level and retrieved as full reports. Search keywords were “Kawasaki disease,” “Kawasaki syndrome,” “lymph node syndrome,” “mucocutaneous lymph node syndrome” combined with “polymorphism,” “gene,” “genetic,” “allele,” and “genotype”. The inclusion criteria of genes were those who have significant association with KD contributed to susceptibility, vascular lesions, resistance to initial IVIG treatment, late diagnosis of KD, and incomplete KD.

### 2.2. Data Analysis

DAVID (http://david.abcc.ncifcrf.gov/, version: 6.7) was used to process the bioinformatics analysis of these candidate gene markers, including gene classification (based on Biological Process Ontology and Molecular Function Ontology, resp.), enrichment analysis for significant gene ontology categories, KEGG (Kyoto Encyclopedia of Genes and Genomes) pathway mapping, and significant pathway computing. GeneGo MetaCore (http://www.genego.com/, version: 6.5) was used to analyze the pathways of these significant genes. The association between these statistically significant genes were analyzed using STRING (http://string-db.org/), a database of known and predicted protein interactions.

## 3. Results

### 3.1. Extracting Genes with Statistical Significance

The characteristics of the genes are presented in Table 1 (candidate genetic studies) and Table 2 (genome-wide studies).

### 3.2. Gene Ontology Analysis

Genes with statistical significance were submitted to functional analysis using DAVID software. Defense response, response to wounding, and inflammatory response were identified as significantly enriched (Enrichment Score = 15.91). Furthermore, DAVID analysis identified clusters of genes with annotations related to cellular calcium ion homeostasis, cell chemotaxis (enrichment Score: 3.75), and positive regulation of immune system process (Enrichment Score: 3.58) which is involved in autoimmune thyroid disease (hsa05320), asthma (hsa05310), type I diabetes mellitus (hsa04940), and allograft rejection (hsa04672). The functional annotation table can be available in supplementary material available online at http://dx.doi.org/10.1155/2013/989307.

### 3.3. Enrichment Analysis

**Figure 1:**
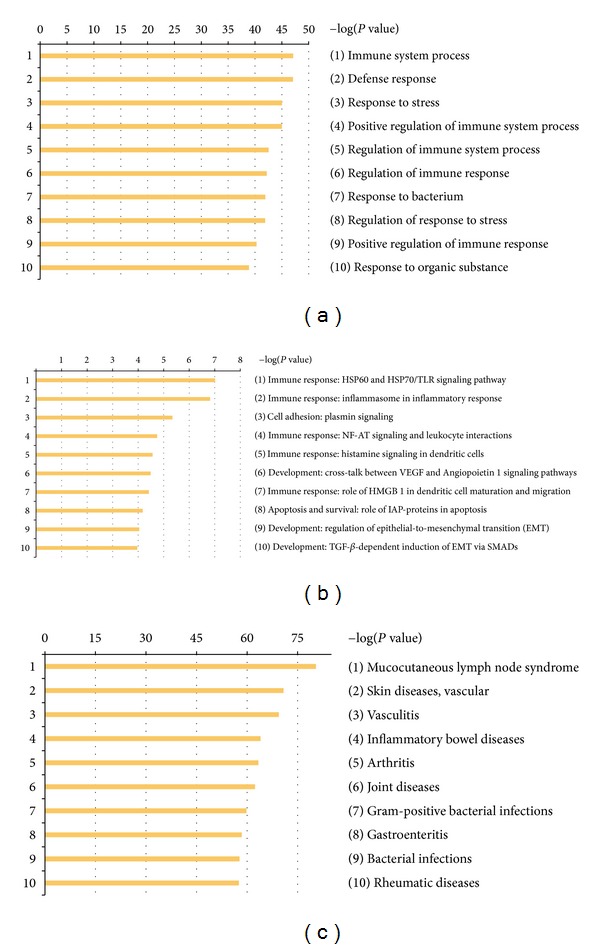
Enrichment analysis of the genes by GeneGo MetaCore: (a) GO Processes, (b) Go Pathway Maps, (c) Go Diseases (by Biomarkers). MetaCore version 6.11 build 41105.

Enrichment analysis consists of matching genes in functional ontologies by GeneGo MetaCore (Figure 1). The probability of a random intersection between a set of gene list with ontology entities was estimated with the “*P*” value of the hypergeometric intersection. A lower “*P*” value means higher relevance of the entity to the dataset, which appears in higher rating for the entity. All maps were drawn by GeneGo. The height of the histogram corresponded to the relative expression value for a particular gene.

The most significant GeneGo Pathway Maps were (1) immune response: HSP60 and HSP70/TLR signaling pathway; (2) immune response: Inflammasome in inflammatory response; (3) cell adhesion: plasmin signaling; (4) immune response: NF-AT signaling and leukocyte interactions. In addition, there are other pathways including Role of HMGB1 in dendritic cell maturation and migration; histamine signaling in dendritic cells: plasmin signaling in cell adhesion; cross-talk between VEGF and angiopoietin 1 signaling pathways; regulation of epithelial-to-mesenchymal transition (EMT); TGF-beta-dependent induction of EMT via SMADs in Development; role of IAP-proteins in apoptosis pathway in apoptosis and survival, and so forth. Meanwhile, immune system process, defense response, and response to stress were the most significantly enriched GO processes of these genes. With the disease folders, representing over 112 human diseases annotated by GeneGo, these 76 genes were mainly related to autoimmune diseases and some kinds of vascular inflammatory diseases.

The abstracted genes involved in significant pathways are summarized in Table 3.

### 3.4. STRING Analysis

Now specifically, we are interested in finding functional associations among these genes. We broadcast our data to STRING (a database of known and predicted protein interactions), which responds by displaying a network of nodes (proteins) connected by colored edges representing functional relationships.

**Figure 2:**
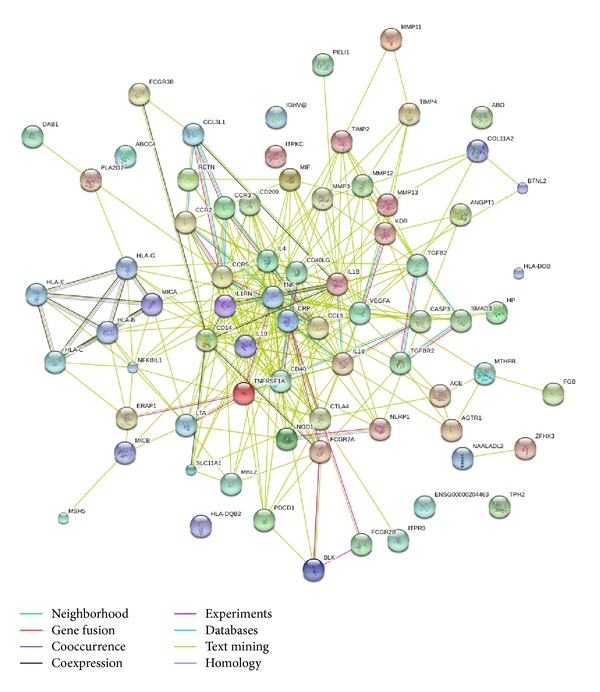
STRING analysis of the relationship between genes. The network nodes represent the proteins encoded by the genes. Seven different colored lines link a number of nodes and represent seven types of evidence used in predicting associations. Among these significant genes, VWA7, PRRC2A, and ABHD16A were not identified. A red line indicates the presence of fusion evidence; a green line represents neighborhood evidence; a blue line represents cooccurrence evidence; a purple line represents experimental evidence; a yellow line represents text mining evidence; a light blue line represents database evidence [100, 101] and a black line represents coexpression evidence.

Figure 2 summarizes the network of predicted associations between proteins encoded by these genes. The results indicate that CASP3, IL18, BLK, FCGR2B, FCGR2A, CRP, CCR5, CCL5, CCR3, CCL3L1, TNFRSF1A, TNF, IL4, ERAP1, LTA, CD40, NOD1, CTLA4, NLRP1, TGFBR2, SMAD3, TGFB2, VEGFA, KDR, and CCR2 are associated according to experimental evidence, with involvement in many signaling pathways; TNF was the key of nodes, linking to CRP, IL-4, CD40, CD40LG, IL-18, IL-10, and so on. They linked to many immune and inflammatory responses. All of these proteins (encoded by genes) are interrelated, forming a large network. However, many proteins are not linked to others, indicating that their functions are unrelated or unknown.

## 4. Discussion

### 4.1. Immune Response in the Pathogenesis of KD

KD has long been considered as an abnormal immune disease. The activation of immune system and the cascade release of inflammatory factors are the important features in KD. A large number of T cells (increased activated CD4 T cells, depressed CD8 T cells and CD4+ CD25+ regulatory T cells), large mononuclear cells, macrophages and plasma cells, with a smaller number of neutrophils, are observed in various organ tissues of fatal cases of acute KD [102–106]. Additionally, various inflammatory cytokines and chemokines [107, 108], matrix metalloproteinases, nitric oxide production [109], autoantibody production [110, 111], and adhesive molecule expression [112, 113] are also overactivated in the acute stage of KD which are considered to facilitate vascular endothelial inflammation and then participate in the pathogenesis of KD and CAL formation. Go processes and DAVID analysis revealed that these genes are significantly enriched in immune responses which have the parallel results with clinical and laboratory findings. In addition, these genes are widely involved in other immune systemic and inflammatory diseases, for example, autoimmune thyroid disease, asthma, type I diabetes mellitus, allograft rejection, inflammatory bowel disease, vasculitis, arthritis, and rheumatic disease. Furthermore, the signal pathway produced in GeneGo contains many immune response pathways that participate in inflammation, apoptosis, injury, and remodeling process, which have been listed in Table 3.

### 4.2. ECM-Remodeling and Plasmin Signaling Pathway in the Pathogenesis of KD

In addition to the signal pathway of the immune response, ECM-remodeling and plasmin signaling pathway associated with cell adhesion were enriched in GeneGo MetaCore software (FDR < 0.01,  *P* < 0.005). 

Numerous studies suggest that they participated in the pathophysiological process of KD. Activation of the fibrinolytic system, vascular injury, and remodeling were the prominent outcome in these pathways. Activated plasmin in the plasmin signaling pathway which is a major fibrinolytic protease can directly degrade fibrinogen, laminin, and fibronectin [114]. On the cell surface, plasmin can activate a number of matrix metalloproteinases (MMPs) MMP1, MMP13 [115]. Other MMPs (MMP-9 and so on) were subsequently activated. Moreover, IL-1 *β*, IL-6, TNF-*α*, and IFN-*γ* can stimulate the endothelial cells to produce more MMP-9. These MMPs degrade extracellular matrix proteins and components of basal membranes leading to the disruption of the internal elastic lamina and the trilaminar structure of the vascular wall [116–118]. Many examinations have showed that many MMPs were highly expressed in the acute stage of KD. MMPs are prominent during the remodeling process, contributing to the formation of coronary artery lesions [119], and consequently the intima proliferates and thickens, while in rare cases the vessel wall becomes stenotic or occluded by either stenosis or thrombosis. Endogenous tissue inhibitors of metalloproteinases (TIMPs) such as TIMP1, TIMP2, and TIMP3 can reduce excessive proteolytic ECM degradation by MMPs. The balance between MMPs and TIMPs controls the extent of ECM remodeling [120, 121]. One study indicated that MMPs and TIMPs were in a state of imbalance in KD patients [122]. Therefore, ECM-remodeling and plasmin signaling pathway may have played a certain role in the vascular damage in KD.

### 4.3. NF-AT Signaling and Leukocyte Interactions

**Figure 3:**
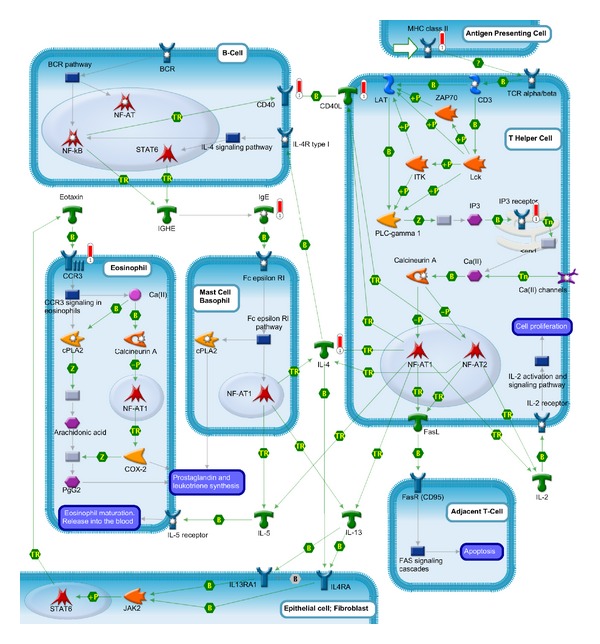
NF-AT signaling and leukocyte interactions have been enriched by GeneGo.

NF-AT signaling and leukocyte interactions (*P* value = 2.28 × 10^−5^) in the immune response cause our great concern. In this pathway, the activation of NFAT proteins is induced by the engagement of receptors that are coupled to the calcium/calcineurin signals, such as the antigen receptors that are expressed by T cells (TCR) and B cells (BCR), the Fc-epsilon receptors (e.g, Fc epsilon R1) that are expressed by mast cells and basophil cells or receptors coupled to heterotrimeric G-proteins (e.g., CCR3 on eosinophils) [123, 124] (Figure 3).

The NFAT signal is activated in T cell and can promote the expression of the immune-related genes. Antigen presenting cells present antigenic peptides to the T helper cell via major histocompatibility complex, class (II) (MHC class II). MHC class II can upregulate the expression of CD4+T cells and downregulate the expression of CD8+T cells which has been confirmed in acute phase of KD. Then, MHC class II peptides activate the T-cell receptor (TCR alpha/beta-CD3 complex) that starts a signal leading to the increase in cytosolic Ca(II) through both the transient release of calcium from intracellular stores and the influx of calcium through Ca(II) channels. That leads to activation of the calcium-regulated phosphatase, Calcineurin A. The activated Calcineurin A cleaves an inhibitory phosphate residue from the transcription fator NF-AT (e.g., NF-AT1 and NF-AT2). Consequently, NF-AT is transported into the nucleus, where it cooperates with other transcription factors for promoter binding and thereby induces the expression of cytokines and many other T-cell-activation-induced proteins. NF-AT in T cells is critical for the expression of a number of immunologically important genes, including IL-2, IL-4, IL-5, and IL-13, as well as several related membrane-bound proteins such as CD40 Ligand (CD40L) and Fas Ligand (Fasl) [125–127].

IL-4 plays an important role in cell-to-cell activation to activate NFAT signal to release leukotrienes and prostaglandins. Activated by NFAT signal in T cell, IL-4 activates nearby B cells that express corresponding receptor, IL-4R. In conjunction with BCR, IL-4 signaling pathway leads to the activation of several transcription factors, including nuclear factor kappa-B(NF-*κ*B), signal transducer, and activator of transcription 6 (STAT6), that regulate immunoglobulin class switching and the production of immunoglobulin E (IgE) by some B cells [128–130]. IgE in turn activates NF-AT1 translocation and function in mast cells and basophils through the IgE receptor (Fc epsilon R1) leading to production of an array of cytokines, including IL-4, IL-5, and IL-13 [131, 132]. Fc epsilon R1 pathway also leads to activation of the cytosolic phospholipase A2 (Cpla2) that contributes to the secretion of leukotrienes and prostaglandins, the main mediators of inflammatory response [133]. IL-4 and IL-13, in turn, activate epithelial cells and/or fibroblasts to release eosinophil-activating cytokines, such as chemokine ligand 11 (Eotaxin). These cytokines recruit eosinophils to the inflammatory focus in the tissue and induce intracellular signaling, mainly via chemokine receptor 3 (CCR3) activation, which leads to the leukotrienes and prostaglandins synthesis and also can use NF-AT1 transcription complex to activate cytokines and chemokines. IL-4 plays an important role in the interaction between the leukocytes and induces the release of variety of inflammatory mediators.

Additionally, CD40L activates nearby B cells that express corresponding receptor CD40. IL-2 binds to IL-2 receptors at the T Cells surface to drive clonal expansion of the activated cell that induces autocrine proliferation [124]. Fasl activates the adjacent T Cells via binding to its receptors; FasR (CD95) [134] mediates apoptosis through the FAS signaling cascades (apoptosis). Fas-Fas ligand system has been considered to be involved in inducing apoptosis in KD resulting in marked decrease of peripheral blood lymphocytes [135]. 

#### 4.3.1. What Is the NFATs?

NFATs are nuclear factors of activated T cells. The NFAT family consists of five members: NFAT1, NFAT2, NFAT3, NFAT4, NFAT4, and NFAT5. Four (except NFAT5) of these proteins are regulated by calcium signaling and four (except NFAT3) are expressed in the immune system [124]. They are initially identified as Ca^2+^-sensitive transcription factors that regulate gene transcription in response to intracellular Ca^2+^signals. NFAT family members are expressed by almost every cell type, including the immune system and nonimmune cells, contributed to the regulation of immune response, as well as development and differentiation. In the immune system, NFATs have pivotal roles in the development and function of immune organs and regulate numerous physiological processes. With the best described effects on T cell activation and phenotype, NFATs also regulate gene expression in other immune cells such as B cells [136], mast cells [137, 138], eosinophils [139], basophils [140] and NK cells [141], macrophage [142], and dendritic cells [143]. They can regulate the release of various cytokines in immune cells. In nonimmune cells, they regulate development and differentiation in a variety of organ systems [134]. It has been examined that they control gene expression during remodeling and are activated by growth factors [144, 145] or histamine [146] in the endothelium, contributing to cell growth, remodeling of smooth muscle cells [147–149], and vascular development and angiogenesis [150–152] (including the isoforms c1 and c3) and are activated in response to inflammatory processes [153] and high intravascular pressure [154] in the vascular system. The isoforms NFATc3 and NFATc4 are active during pathophysiological conditions that affect the cardiovascular system, including atrial fibrillation [155, 156] and hypertrophy [157]. Loss of specific NFAT isoforms has been found to result in cardiovascular, skeletal muscle, cartilage, neuronal, or immune system defects [158–162]. Therefore, we can conclude that the Ca^2+^/NFAT pathway plays a wide range role in inflammatory processes, immune responses, and the remodeling of vascular tissues. All of these physiological processes occur in KD. It is suggested that the Ca^2+^/NFAT pathway may involve in the pathological processes of KD.

#### 4.3.2. The Upstream Adjustment Signals of NFAT Signal

NFATs are mainly Ca^2+^-sensitive transcription factors that regulate gene transcription in response to intracellular Ca^2+^ signals. Four (except NFAT5) of these proteins are regulated by calcium signaling. Activity of NFATs is regulated by phosphorylation. Inactive NFATs are highly phosphorylated and localized in the cytoplasm. Intracellular Ca^2+^ signals activate the calmodulin-dependent serine/threonine phosphatase calcineurin (CaN), which dephosphorylates NFATs and induces translocation to the nucleus.

Inositol-trisphosphate 3-kinase C (ITPKC) is a negative regulator of the Ca^2+^/NFAT pathway. NF-AT signaling was first mentioned to be associated with regulation of ITPKC in the KD. ITPKC is a kinase of inositol 1,4,5-triphosphate (IP3) which is a second messenger molecule that releases calcium from the endoplasmic and sarcoplasmic reticulum. First identified by genome-wide study and following confirmation by candidate genetic studies in both Japanese, Taiwanese and US children, ITPKC was considered to be associated with KD which confers both susceptibility to KD and the risk for CAL and IVIG resistance [26, 94, 95, 163], which has been thought to be involved in the Ca^2+^-dependent NFAT signaling pathways in T cells. It has been considered that C allele of rs28493229 in ITPKC can reduce the splicing efficiency of the ITPKC mRNA, inducing the hyperactivation of Ca^2+^-dependent NFAT signal in T cells, leading to a reduction in the phosphorylation of IP3 to IP4, resulting in the increase of IP3 levels. This would result in an increase of calcium levels and excessive activation of the NFAT signal, thus leading to immune dysregulation.

Caspase-3 (CASP3) is a key molecule of activation-induced cell death (AICD) [164]; it is profoundly related to the apoptosis of immune cells. It has also been reported to cleave the inositol 1,4,5-triphosphate receptor, type 1 (ITPR1) in apoptotic T cells (ITPR1 is a receptor for inositol 1,4,5-trisphosphate (IP3), a substrate for ITPKC in T cells [165]). Thereby, it is a positive regulatory factor of NFAT signal. Additionally, the mutation of CASP3 (rs113420705) can reduce the binding of NFAT to the DNA surrounding the SNP. Its gene variant (4q34-35, rs113420705) has been identified contributing to KD susceptibility in Euro-American triads and Taiwanese [35, 166]. Other studies [167, 168] also stated that CASP3 plays an important role in the execution phase of apoptosis of immune cells in KD. 

Calcineurin inhibitors (e.g., CsA, FK506) have been extensively used as immunosuppressive agents to improve graft survival and to treat autoimmune diseases [127]. They act by blocking calcineurin enzymatic activity. CsA has been an effective [169–171] therapeutic drug in the treatment of IVIG resistance patients in KD. 

#### 4.3.3. The Downstream of Adjustment Signals of NFAT Signal: NF-*κ*B (Nuclear Factor Kappa-B)

NF-*κ*B is another transcription factor of eukaryotes, which is evolutionarily related to the NF-AT family of transcription factors. It is activated in response to signals that lead to cell growth, differentiation, apoptosis, and other events. It takes part in expression of numerous cytokines and adhesion molecules which are critical elements involved in the regulation of immune responses. 

NF-*κ*B plays pivotal roles in the immune and inflammatory responses by regulating the interaction between CD40 and CD40L in T cells and B cells. NF-*κ*B can be activated by IL-4 signaling pathway in B cells to induce the expression of CD40 which has been illustrated above. CD40 plays a crucial role as a costimulatory molecule in the cooperation between T and B cells. It is important in the pathogenesis of autoimmune diseases in humans and animal models such as autoimmune thyroiditis, inflammatory bowel disease, psoriasis, systemic lupus erythematosus, allergic encephalomyelitis, multiple sclerosis, rheumatoid arthritis, collagen-induced arthritis, and autoimmune type of diabetes mellitus [172–174]. CD40 signaling leads to isotype switching and autoantibody production in B cells and in T-cell priming, altering TCR expression through the expression and nuclear translocation of recombinases, which increases the risk of developing autoimmunity [173]. CD40 engagement in both T or B cells leads to the production of cytokines, such as IL-12, IL-2, TNF-*α*, IFN-*α*, and CD80, developing an environment which is conducive to autoimmune diseases [172–174].

Additionally, the interaction between CD40 and CD40L regulated by NF-*κ*B can regulate the expression of numerous biomolecules in other cells. They can enhance the expression of cytokines (such as IL-2, IL-6, IL-10, TNF-*α*, lymphotoxin-*α*, and transforming growth factor-*β* by B cells; the synthesis of granulocyte macrophage colony-stimulating factor (GM-CSF) by dendritic cells and eosinophils and the synthesis of TNF-*α*, IL-1, IL-6, and IL-8 by peripheral blood mononuclear cells), chemokines (monocyte chemotactic protein-1 (MCP-1), IL-8, MCP-1, matrix metalloproteinases (MMP-1,-2,-3,-9,-11, and -13) by peripheral blood mononuclear cells, macrophages, endothelial and smooth muscle cells endothelial), adhesion molecules (E-selectin, vascular cell adhesion molecule-1 (VCAM-1) and intercellular adhesion molecule-1 (ICAM-1) in endothelial cells and fibroblasts), platelet-activating factor [175], prostaglandin E2 [176], vascular endothelial growth factor [177, 178], and NO [172]. which are involved in the pathophysiology of inflammatory and autoimmune diseases.

NF-*κ*B may participate in the pathogenesis of vasculitis of KD in acute stage. Some studies have indicated that NF-*κ*B is excessively activated in the acute phase of KD and the inhibition of NF-*κ*B can reduce the generation of inflammatory cytokines which plays important roles in vascular damage of KD [179, 180]. NF-*κ*B signaling pathway is a complex system; it perhaps involves in immune damage of KD in different levels. Activation of NF-*κ*B can be used as the trigger of key links of the inflammatory response and induce the cascade release of inflammatory response factor, eventually leading to inflammatory pathological damage.

### 4.4. The NF-AT Signaling and Leukocyte Interactions and NF-*κ*B Signaling Together May Be Involved in the Pathogenetic Process of KD

Given the important role of NFAT signaling and NF-*κ*B signaling in the activation of immune system and the regulating of vascular remodeling, we speculate that the interaction between NFAT signaling and NF-*κ*B signaling together may also be involved in the pathogenesis of KD. 

Initially due to exposure to some inflammatory stimuli or certain pathogens, antigen presenting cells present antigenic peptides to the T-cell receptors via MHC class II leading to the stimulation of PLC-gamma 1 and hydrolyzation of PIP2. The second messengers IP3 in the T cells start a signal leading to the increase in cytosolic Ca(II) through both the transient release of calcium from intracellular stores and influx of calcium through Ca(II) channels. The high calcium levels lead to activation of the calcium-regulated phosphatase, Calcineurin A. The activated Calcineurin A cleaves an inhibitory phosphate residue from the transcription fator NF-AT (e.g., NF-AT1 and NF-AT2). Consequently, NF-AT is transported into the nucleus, where it cooperates with other transcription factors for promoter binding and activates T cells inducing the expression of a number of immunologically important genes including IL-2, IL-4, IL-5, IL-13, CD40 Ligand (CD40L), and Fas Ligand (Fasl). Through the leukocyte interactions, other immune cells were activated and release other inflammatory cytokines, such as leukotrienes and prostaglandins. In B cells and T-cell, CD40 signaling leads to isotype switching, autoantibody production, and altering TCR expression. CD40 signaling can also enhance the expression of cytokines, chemokines, matrix metalloproteinases, adhesion molecules, platelet-activating factors, prostaglandin E2, vascular endothelial growth factor, and NO. in other cells. The combined effect of these factors causes the vascular damage and formation of coronary artery lesions in KD. The process of NFAT signal in regulating development and differentiation was also excessively induced by the pathological damage of vasculature and then contributed to the remodeling of vascular system.

IL-4, CD40, and CD40L, which are enriched in the pathway of NF-AT signaling and leukocyte interactions and play a crucial role in the immune response and remodeling process, are located in the center position of the network (analysed by STRING) and are closely linked with the other factors. It further demonstrate the importance of this pathway.

## 5. Conclusions

KD is a complex disease. Many studies have shown that it is associated with a variety of gene polymorphism. Through GeneGo and DAVID analysis, we speculated that NF-AT signaling and leukocyte interactions combined with another transcription factor NF-*κ*B may play an important role in pathological damage of KD. Their importance needs our follow-up clinical validation.

## Supplementary Material

The Supplementary Material was made by the software of DAVID (The Database for Annotation, Visualization and Integrated Discovery). It provides a comprehensive set of functional annotation tools for investigators to understand biological meaning behind large list of genes. The Supplementary Material contains the gene ontology analysis and the pathway analysis (KEGG pathway) of my genes which has been presented in Table 1 (candidate genetic studies), Table 2 (genome-wide studies). Gene ontology analysed the gene function from molecular function, biological process, cellular component. KEGG pathway showed the biological pathways and diseases these genes involved in.

## Figures and Tables

**Table 1:** Candidate gene studies identified genes associated with KD.

Symbol	Region	Phenotype	Country	Reference
CD40	20q12-q13.2	KD	Taiwan	[20]
		CAL	Taiwan	[20]
CD209	19p13	KD	Taiwan	[21]
RETN	19p13.2	Incomplete KD	China	[22]
		KD	United States	[23]
		CAL	Japan	[24]
FCGR3B	1q23	IVIG nonresponse	United States	[23]
NOD1	7p15-p14	KD	Japan	[25]
NLRP1	17p13.2	KD	Japan	[25]
ITPKC	19q13.1	KD	Taiwan; Japan; China	[26–28]
		CAL	Japan	[29]
		IVIG nonresponse	Japan	[29]
TGFBR2	3p22	KD	European descent; Korea	[30, 31]
		CAL	European descent; Korea	[30, 31]
		IVIG nonresponse	European descent	[30]
		aortic root dilatation,	European descent	[30]
ABO	9q34.2	CAL	Japan	[32]
PELI1	2p13.3	CAL	Korea	[33]
SMAD3	15q22.33	KD	European descent; Taiwan	[30, 34]
		CAL	European descent	[30]
		IVIG nonresponse	European descent	[30]
		aortic root dilatation	European descent	[30]
TGFB2	1q41	KD	European descent; Taiwan	[30, 34]
		CAL	European descent	[30]
		IVIG nonresponse	European descent	[30]
		aortic root dilatation,	European descent	[30]
CASP3	4q34	CAL	Taiwan; Japan	[29, 35]
		IVIG nonresponse	Japan	[29]
ANGPT1	8q23.1	KD	Netherlands	[36]
VEGFA	6p12	KD	Netherlands; Taiwan; The Netherlands.	[36–38]
		CAL	Japan	[39]
MICB	6p21.3	KD	Taiwan	[40]
		CAL	Taiwan	[40]
MICA	6p21.33	KD	Taiwan	[40]
		CAL	Taiwan	[41]
BAG6	6p21.3	KD	Taiwan	[40, 42]
		CAA	Taiwan	[42]
MSH5	6p21.3	KD	Taiwan	[40]
VWA7	6p21.33	KD	Taiwan	[40]
FCGR2B	1q23	IVIG nonresponse	Pacific Northwest	[43]
IL10	1q31-q32	KD	Taiwan	[44, 45]
		CAL	China; Korea; Taiwan	[18, 46, 47]
CCL5	17q11.2-q12	CAL	India	[48]
TNFRSF1A	12p13.2	KD	China	[49]
CTLA4	2q33	CAL (particularly in female patients)	Taiwan	[50]
MMP3	11q22.3	CAL	Korea; US-UK, tested in Japan	[51, 52]
MMP12	11q22.3	CAL	US-UK, tested in Japan	[52]
FGB	4q28	CAL	China	[53]
CCL3L1	17q21.1	KD	USA; Japan	[54, 55]
		IVIG nonresponse	Japan	[55]
CCR5	3p21.31	KD	USA; The Netherlands (Dutch Caucasian); Korea	[54, 56, 57]
		CAL	Japan	[55]
		IVIG nonresponse	Japan	[55]
PRRC2A	6p21.3	KD	Taiwan	[42]
		CAL	Taiwan	[42]
ABHD16A	6p21.3	KD	Taiwan	[42]
		CAL	Taiwan	[42]
ITPR3	6p21	CAL	Taiwan	[58]
COL11A2	6p21.3	KD	Taiwan	[59]
		CAL	Taiwan	[59]
MBL2	10q11.2	KD	China; Japan	[60, 61]
		CAL	The Netherlands; The Netherlands	[62, 63]
		Arterial stiffness	China	[64]
MMP11	22q11.23	KD	Korea	[65]
MIF	22q11.23	CAL	Italy	[66]
IL1B	2q14	IVIG nonresponse	Taiwan	[17]
BTNL2	6p21.3	KD	Taiwan	[67]
		CAL	Taiwan	[67]
TPH2	12q21.1	CAL	Korea	[68]
PDCD1	2q37.3	KD	Korean	[69]
IL18	11q22.2-q22.3	KD	Taiwan	[70, 71]
HLA-E	6p21.3	KD	Taiwan	[72]
		CAL	Taiwan	[72]
TIMP4	3p25	CAL	Korea	[73]
HLA-G	6p21.3	KD	Korea	[74]
CRP	1q21-q23	KD	China	[75]
		Carotid stiffness and carotid intima-media thickness	China	[75]
TNF	6p21.3	KD	China	[75]
		CAL	white	[76]
		Intima-media thickness	China	[75]
		IVIG nonresponse	China	[46]
MMP13	11q22.3	CAL	Japan	[77]
HLA-B	6p21.3	KD	Korea	[78]
HLA-C	6p21.3	KD	Korea	[78]
CCR3	3p21.3	KD	Netherlands (Dutch Caucasian)	[56]
CCR2	3p21.31	KD	Netherlands (Dutch Caucasian)	[56]
TIMP2	17q25	CAL	Japan	[79]
ACE	17q23.3	KD	Taiwan; Korea	[80, 81]
		Coronary artery stenosis	Japan	[82, 83]
		Myocardial ischemia	Japan	[82]
PLA2G7	6p21.2-p12	IVIG nonresponse	Japan	[84]
IL1RN	2q14.2	KD	Taiwan	[85]
IL4	5q31.1	KD	USA	[86]
KDR	4q11-q12	CAL	Japan	[39]
CD40LG	Xq26	CAL: males affected	Japan	[87]
AGTR1	3q24	Coronary artery stenosis and myocardial ischemia	Japan	[82]
CD14	5q31.1	CAL	Japan	[88]
SLC11A1	2q35	KD	Japan	[89]
LTA	6p21.3	KD	white	[76]
MTHFR	1p36.3	CAL	Japan	[90]
HP	16q22.2	late diagnosis of KD	Taiwan	[90]

KD: kawasakidisease;CAL: coronary artery lesions; CAA: coronary artery aneurysms.

**Table 2:** Susceptibility genes for KD identified with association at genome-wide significance.

Gene	Locus	Methods	Reference
FCGR2A	1q23	GWAS	[91]
BLK	8p23-p22	GWAS	[92, 93]
CASP3	4q34	Genome wide Linkage analysis	[94]
ITPKC	19q13.1	Genome wide Linkage analysis; linkage disequilibrium mapping	[94, 95]
CD40L	Xq26	Genome wide Linkage analysis	[94]
CD40	20q12-q13.2	GWAS	[92, 93]
HLA-DQB2	6p21	GWAS	[92]
HLA-DOB	6p21.3	GWAS	[92]
NFKBIL1	6p21.3	GWAS	[92]
LTA	6p21.3	GWAS	[92]
NAALADL2	3q26.31	GWAS	[96]
ZFHX3	16q22.3	GWAS	[96]
DAB1	1p32-p31	GWAS	[97]
PELI1	2p13.3	GWAS	[97]
COPB2	3q23	GWAS	[98]
ERAP1	5q15	GWAS	[98]
IGHV	14q32.33	GWAS	[98]
ABCC4	13q32	Genome-wide linkage and association mapping	[99]

GWAS: genome-wide association study.

**Table 3:** Pathways analyzed by GeneGo Meta core.

Pathway categories	Pathways	Functions	Enrichment genes
Immune response	(1) HSP60 and HSP70/TLR signaling pathway (2) Inflammasome in inflammatory response (3) NF-AT signaling and leukocyte interactions (4) Role of HMGB1 in dendritic cell maturation and migration (5) Histamine signaling in dendritic cells (6) CD16 signaling in NK cells (7) MIF in innate immunity response (8) Th1 and Th2 cell differentiation (9) HMGB1 release from the cell (10) PGE2 signaling in immune response (11) Histamine H1 receptor signaling in immune response (12) Role of DAP12 receptors in NK cells	Pro-inflammatory response and anti-inflammatory response; cellular and humoral immune response; NO production; apoptosis and antiapoptosis; secretion of leukotrienes and prostaglandins; proliferation and differentiation of eosinophils; chemotaxis; proliferation, differentiation, activation of T cell; cell necrosis; smooth muscle construction; vascular permeability; blood coagulation; cytoskeleton remodeling	CD14, HSP70, IL-10, TNF-*α*, IL-1*β*, CD40, MHC class I, IL-4, NOD1, CARD7, IL-18, TNF-R1, CD40L, IP3receptor, CCL5, HLA-E, PLA2, MIF, CCR5, MMP13, HLA-C, HLA-B, HLA-G, HLA-E, Stromelysin-1

Cell adhesion	Plasmin signaling ECM remodeling	Fibrinolysis; cell viability	TGF-*β* 2,VEGF-A, TGF-*β* receptor type 2, VEGFR-2, Fibrinogen, MMP-13, TIMP2, Stromelysin-1, MMP-13, MMP-12

Development	(1) Cross-talk between VEGF and Angiopoietin 1 signaling pathways (2) Regulation of epithelial-to-mesenchymal transition (EMT) (3) TGF-*β*-dependent induction of EMT via SMADs (4) PEDF signaling (5) Glucocorticoid receptor signaling	Leukocyte-endothelial adhesion; epithelial-to-mesenchymal transition; proteasomal degradation; inhibition of angiogenesis; immune response	VEGF-A, VEGFR-2, Angiopoietin 1, IP3 receptor, TGF-*β* 2, IL-1*β*, TNF-*α*, TNF-R1, TGF-*β* receptor type 2, SMAD3, TGF-*β*, HSP70, MMP13

Apoptosis and survival	(1) Role of IAP-proteins in apoptosis (2) Anti-apoptotic TNFs/NF-kB/Bcl-2 pathway	Caspase dependent and independent apoptosis; apoptosis and antiapoptosis	TNF-*α*, TNF-R1, HSP70, caspase3, CD40L, CD40

Transcription	NF-kB signaling pathway	Activate the transcription of target genes	TNF-*α*, TGF-*β*, TNF-R1, CD14

FDR = 0.01.
